# A Novel Competing Endogenous RNA Network Reveals Potential Mechanisms and Biomarkers of Chemoresistance in Lung Adenocarcinoma

**DOI:** 10.7150/jca.102148

**Published:** 2025-01-01

**Authors:** Weisha Liu, Hanxiao Zhou, Yue Qi, Peng Wang, Shangwei Ning, Yue Huang, Liuying Wang, Lei Cao, Kang Li

**Affiliations:** 1 Department of Biostatistics, School of Public Health, Harbin Medical University, Harbin 150081, China.; 2 Institute of Cancer Prevention and Treatment, Harbin Medical University, Harbin 150081, China.

**Keywords:** ceRNA, lncRNA, chemoresistance, lung adenocarcinoma, bioinformatics

## Abstract

Platinum resistance is a common cause of chemotherapy failure in lung adenocarcinoma (LUAD). Competing endogenous RNAs (ceRNAs), which function by competitively binding to miRNAs, can influence drug response. However, the regulatory mechanisms of ceRNAs underlying chemoresistance in LUAD remain largely unknown. Here, we proposed an integrated pipeline combining limma algorithm, miRNA binding prediction algorithm, expression correlation model and experimental support to identify functional lncRNA-miRNA-mRNA competing triplets associated with resistance, which showed variable competing patterns between resistant and sensitive cells. We found that a minority of altered ceRNAs overlapped in multiple types of cisplatin-resistant LUAD cell lines and were involved in biological processes known to mediate cancer drug response. We identified them as core resistance factors, forming a novel lncRNA-mediated resistance-related ceRNA network, which indicated a potential mechanism. Single-cell analysis revealed that these resistance-related ceRNAs regulated the functional states of LUAD cells, and survival analysis showed that they contributed to the prognosis of LUAD patients. The lncRNA regulators *H19* and *MIR193BHG* were found to correlate with cisplatin activity in LUAD cell lines, and dysregulation of their expression triggered disorders of cisplatin response-related functions through multiple ceRNA regulatory axes in this network, suggesting them as potential resistance biomarkers and therapeutic targets. In summary, the integrated pipeline and the resulting data serve as a valuable resource for understanding the ceRNA mechanisms of chemoresistance and improving chemotherapy response.

## Introduction

Lung adenocarcinoma (LUAD) is the major pathologic subtype of non-small cell lung cancer (NSCLC) and has a poor 5-year survival rate 1, 2. Since LUAD is usually diagnosed at an advanced stage, most patients require systemic chemotherapy. Platinum compounds, such as cisplatin (CDDP), are first-line chemotherapeutic agents for LUAD 3. However, the efficacy of chemotherapy is severely limited by the emergence of drug resistance. There is increasing evidence that the mechanisms of CDDP resistance are complex and multifactorial 4, 5. Several factors (including protein-coding and non-coding RNAs) have been proposed to contribute to CDDP resistance by affecting hallmarks of drug resistance, such as increased DNA damage repair, altered proliferation, and dysregulation of drug transport 6-8. However, currently used CDDP-based chemotherapy combination strategies are far from perfect for LUAD patients due to limited efficacy, and there are almost no available methods to effectively overcome CDDP resistance. Therefore, it is imperative to elucidate novel mechanisms of CDDP resistance, and develop novel resistance biomarkers and therapeutic targets to improve the efficacy of LUAD treatment.

Long non-coding RNAs (lncRNAs), a class of transcripts more than 200 nucleotides in length, exert their functions through multiple mechanisms, such as influencing chromatin modification, controlling transcription and translation, as well as competing with mRNAs for microRNA (miRNA) binding 9, 10. Some lncRNAs containing miRNA response elements (MREs) can act as competing endogenous RNAs (ceRNAs) to regulate the expression of protein-coding messenger RNAs (mRNAs) by adsorbing miRNAs to relieve miRNA-mediated target repression 11, 12. Rather than being isolated from each other, genes often interact closely when they function. Emerging evidence has demonstrated that lncRNA-mediated ceRNA interactions are not only of fundamental importance in physiological conditions, but also relevant to the pathogenesis of various cancers 13, 14 and may also influence cancer therapy 15-17. Despite advances in ceRNAs, their potential roles in chemoresistance in LUAD are not fully understood.

With the development of pharmacogenomics, the accumulation of a large amount of cancer molecular profiling and drug response data has facilitated the exploration of the mechanism of chemoresistance. In the present study, we explored chemoresistance in LUAD from the perspective of the ceRNA system. To identify functional ceRNAs associated with drug resistance, we proposed a multi-step pipeline combining a computational framework and experimental support (the workflow diagram is shown in Supplementary Fig. S1). We constructed a novel lncRNA-mediated ceRNA network consisting of core lncRNA-miRNA-mRNA competing triplets associated with CDDP resistance in LUAD, which we further validated by independent datasets. We comprehensively analyzed the ceRNA network and reviewed reliable publications. These results demonstrated how ceRNAs could be used as resistance biomarkers and therapeutic targets. This study provides new insights into the ceRNA mechanisms underlying chemoresistance and aids in improving the benefit of cancer chemotherapy.

## Materials and Methods

### Data collection and preprocessing

We obtained gene expression profiles (GSE43493, GSE108139) 18, 19 and miRNA expression profiles (GSE43249, GSE84200) 18, 20 of CDDP-resistant LUAD cell lines (A549R, H23R) and matched CDDP-sensitive LUAD cell lines (A549S, H23S) from the Gene Expression Omnibus (GEO) database. Using the platform annotation file, the probes were matched to gene symbols. The unmatched probes and one-to-many probe-gene matches were removed. When different probes were mapped to the same gene, the mean value of these different probes was used as the final expression of this gene. Bulk RNA-seq-based gene expression profiling data of 535 LUAD tumor tissue samples and corresponding clinical information (specifically, overall survival time) of LUAD patients were obtained from The Cancer Genome Atlas (TCGA) project. Gene expression was transformed into Transcripts Per Kilobase of exon model per Million mapped reads (TPM) format to eliminate the effects of sequencing depth and gene length. In this study, all expression data were log2-transformed. The gene expression profiles were divided into lncRNA and mRNA expression according to the gene annotations in GENCODE 21.

We obtained human lncRNA and mRNA sequences from GENCODE, and human mature miRNA sequences from miRBase 22. Experiment-supported miRNA-lncRNA interactions were obtained from starBase (v3.0) database 23, which provides Argonaute (AGO) crosslinking immunoprecipitation sequencing (CLIP-seq) experiment data. Experimentally supported miRNA-mRNA interactions were obtained from two high-quality miRNA target databases, TarBase (v8) 24 and miRTarBase (v9.0) 25, which provide manually curated collections of miRNA targets supported by Luciferase reporter assay, Western blot assay, qRT-PCR, etc.

We obtained EXP0066 (GSE69405) and EXP0067 (GSE85534) single-cell datasets concerning LUAD from the cancer single-cell state atlas (CancerSEA) database 26, which contained cancer-related scRNA-seq data and gene signatures of 14 functional states of cancer cells, including angiogenesis, proliferation, invasion, metastasis, apoptosis, cell cycle, differentiation, DNA damage, DNA repair, EMT, hypoxia, inflammation, quiescence and stemness. These gene signatures were compiled by hand from public databases (such as MSigDB, HCMDB, Cyclebase) and relevant publications.

We downloaded CDDP activity data, measured by the half-maximal inhibitory concentration (IC50), from the largest public pharmacogenomics database, the Genomics of Drug Sensitivity in Cancer (GDSC), and corresponding bulk RNA-seq-based gene expression data of LUAD cell lines from the Cancer Cell Line Encyclopedia (CCLE). Ultimately, data from 48 common LUAD cell lines with no missing values in the GDSC2 and CCLE datasets were used for further analysis.

### Genome-wide identification of dysregulated molecules

We applied linear models and classical Bayesian test as implemented in the limma R package 27 to examine molecular expression differences between CDDP-resistant LUAD cell lines (A549R, H23R) and matched CDDP-sensitive LUAD cell lines (A549S, H23S). In the preliminary screening, more direct evidence of statistical significance can provide more information to help us detect possible differences. Therefore, lncRNAs and mRNAs with *P* < 0.05 and fold change (FC) > 1.5, and miRNAs with *P* < 0.05, were considered to be significantly differentially expressed. Next, the overlap of differentially expressed molecules with consistent expression patterns between two types of LUAD cell lines (A549 and H23) were identified as candidate dysregulated molecules potentially associated with CDDP resistance in LUAD for further analysis, including dysregulated lncRNAs (DlncRNAs), miRNAs (DmiRNAs) and mRNAs (DmRNAs).

### Identification of functional lncRNA-miRNA-mRNA competing triplets associated with drug resistance

In the current study, we systematically identified the competitive crosstalk among RNA transcripts involved in drug resistance. First, we predicted miRNA binding sites on the whole lncRNA sequences using four computational methods, including TargetScan, miRanda, PITA and RNAhybrid with default parameters 28. Next, the predicted miRNA-lncRNA interactions were further filtered using AGO-CLIP-seq experiment-supported interaction data in starBase (v3.0) database. The intersections with starBase (v3.0) were selected as candidate miRNA-lncRNA interactions. We obtained high-confidence miRNA-mRNA interactions from TarBase (v8) and miRTarBase (v9.0), validated by strong experimental methods. The candidate DlncRNAs, DmiRNAs, DmRNAs were mapped to the miRNA-lncRNA interaction dataset and miRNA-mRNA interaction dataset. Based on the principle of miRNA-binding interaction, only DmiRNA-DlncRNA/DmRNA interaction pairs whose two molecules were dysregulated molecules with expression levels that changed in opposite directions in CDDP-resistant LUAD cells were identified as active interactions for further analysis.

Then, active DlncRNA-DmiRNA and DmRNA-DmiRNA interactions sharing the same DmiRNA were merged into a DlncRNA-DmiRNA-DmRNA triplet. According to the ceRNA mechanism, the expression of lncRNA and mRNA in a ceRNA interaction is positively correlated with each other. Thus, based on gene expression data of LUAD tumor samples obtained from the TCGA project, functional ceRNA interactions were further identified by evaluating the co-expression correlation of lncRNA-mRNA pairs. For each candidate DlncRNA-DmRNA pair, the expression values of lncRNA *i* and mRNA *j* across *n* LUAD tumor samples were defined as 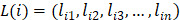 and 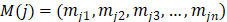, respectively. Since malignant solid tumor tissues consist not only of tumor cells but also normal cells associated with the tumor microenvironment, we calculated the tumor purity for each tumor sample based on the ESTIMATE algorithm, which uses gene expression signatures to infer the proportion of stromal and immune cells in the tumor sample 29. The tumor purity scores across *n* tumor samples were defined as 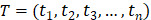. Then, a co-expression correlation model based on tumor sample data was developed to calculate the partial correlation coefficient (PCC) between the expression of lncRNA *i* and mRNA *j*, by considering tumor purity as a co-variable. The detailed formula was displayed as follows:



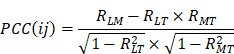



Where 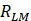 , 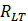 and 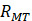 are the Pearson correlation coefficients between the expression of lncRNA *i* and mRNA *j*, the expression of lncRNA *i* and tumor purity, and the expression of mRNA *j* and tumor purity, respectively. In addition, we obtained the p-value for the PCC, defined as 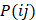. The DlncRNA-DmRNA pairs with correlation PCC > 0.2 and *P* < 0.05 were considered to be co-expressed pairs.

DlncRNA and DmRNA interacting with a common DmiRNA and co-expressed, was eventually identified as a functional DlncRNA-DmiRNA-DmRNA competing triplet associated with CDDP resistance in LUAD.

### Construction of drug resistance-related ceRNA network

This study investigated drug resistance using a ceRNA system that depends on competition for miRNA binding between diverse RNA species. After assembling all candidate functional DlncRNA-DmiRNA-DmRNA competing triplets, a CDDP resistance-related ceRNA network was constructed in LUAD. This network was visualized using Cytoscape software, where nodes represent DlncRNAs, DmiRNAs and DmRNAs, and edges represent regulatory relationships between them.

### Biological function analysis

We performed Gene Ontology (GO) functional annotation and enrichment analysis on downstream target mRNAs in ceRNA interactions to investigate the functional roles of upstream lncRNA regulators and the ceRNA network, using the Database for Annotation, Visualization and Integrated Discovery (DAVID) 30, which is an online analysis tool with the human genome as a reference set and Fisher's exact test to calculate statistical significance.

### Analysis of cancer cell functional states at single-cell level

We used cancer-related scRNA-seq data and gene signatures of 14 cancer cell functional states obtained from CancerSEA database to investigate the correlation of the ceRNA network with the functional states of LUAD cells. Based on the corresponding signatures, the scores of functional state activities across cancer single cells in LUAD single-cell datasets were evaluated by Gene Set Variation Analysis (GSVA) using the GSVA package in R 31, followed by Spearman's rank correlation test to determine the correlations between the expression of downstream target mRNAs in the ceRNA network and the functional state activities of cancer cells (correlation R > 0.3 and *P* < 0.05).

### Survival analysis

We collected clinical follow-up information on LUAD patients from the TCGA project for survival analysis. Patients without overall survival data were excluded, and 522 LUAD samples were ultimately retained. A risk score model was developed to evaluate the association between ceRNA interaction and survival, which took into account both the strength of association and the positive/negative association between the expression of each molecule in the lncRNA-mRNA competing interaction pair and overall survival time. For each LUAD sample, the integrated risk score of ceRNA interaction was calculated based on the linear combination of lncRNA and mRNA expression values weighted by Cox regression coefficients, as follows:



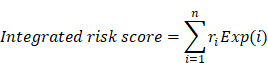



where 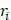 is the Cox regression coefficient of molecule *i* in the ceRNA interaction, from the univariate Cox regression analysis that evaluated the association between the expression of molecule *i* and overall survival time, *n* is the number of molecules in the ceRNA interaction, 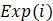 is the expression value of molecule *i* in corresponding tumor sample.

Further, the median integrated risk score for all tumor samples was used as a cut-off to classify samples into two groups. Kaplan-Meier survival analysis was performed on the two groups, and statistical significance was assessed using the log-rank test, *P* < 0.05.

### Drug activity analysis

GDSC CDDP IC50 values and CCLE lncRNA expression data of LUAD cell lines were integrated for investigation. IC50 is an important indicator of the anti-tumor activity of a drug, and a higher IC50 value indicates a lower sensitivity of tumor cells to the drug. Pearson correlation analysis was carried out to assess the correlation between the expression of upstream lncRNAs in the ceRNA network and CDDP IC50 value (correlation R > 0.3 and *P* < 0.05). The positive correlation implies that lncRNA high expression is potentially resistant to CDDP, and vice versa.

## Results

### Identification of dysregulated molecules in CDDP-resistant LUAD cells

**Figure 1:**
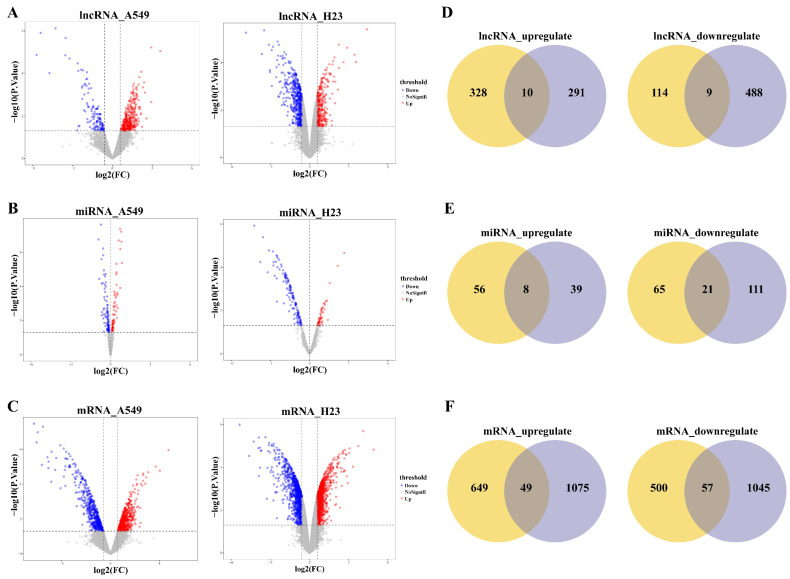
Identification of dysregulated molecules in CDDP-resistant LUAD cells. **(A-C)** Volcano plots show differentially expressed lncRNAs, miRNAs, mRNAs between CDDP-resistant LUAD cell lines (A549R, H23R) and matched CDDP-sensitive LUAD cell lines (A549S, H23S). **(D-F)** Venn diagrams show the overlap of differentially expressed molecules between A549 and H23 cell line, separately for upregulated and downregulated lncRNAs, miRNAs, mRNAs in both A549R and H23R resistant cell lines. Yellow circle represents A549R cell line, purple circle represents H23R cell line.

To investigate the molecular changes contributing to CDDP resistance in LUAD, we used the limma algorithm to screen differentially expressed lncRNAs, miRNAs, mRNAs between CDDP-resistant LUAD cell lines (A549R, H23R) and matched CDDP-sensitive LUAD cell lines (A549S, H23S) (Fig. 1A-C). A total of 19 DlncRNAs, 29 DmiRNAs and 106 DmRNAs were identified as candidate dysregulated molecules potentially associated with CDDP resistance in LUAD for further study, which were overlapping and had consistent expression patterns between A549 and H23 cell lines. These included 10 upregulated and 9 downregulated lncRNAs, 8 upregulated and 21 downregulated miRNAs, 49 upregulated and 57 downregulated mRNAs in both A549R and H23R resistant cell lines (Fig. 1D-F).

**Figure 2:**
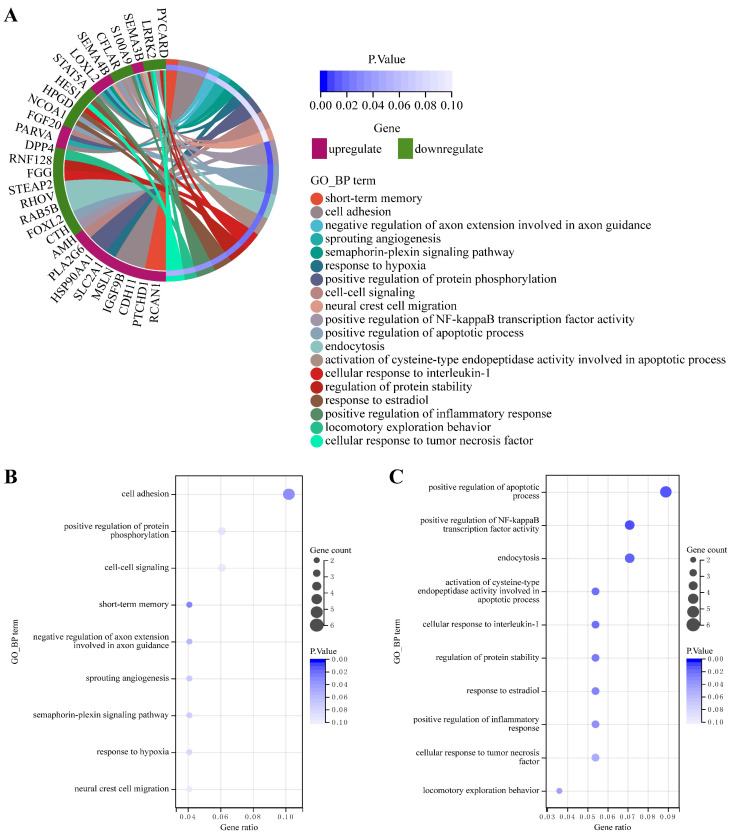
GO functional enrichment of DmRNAs in CDDP-resistant LUAD cells, separately for upregulated and downregulated mRNAs. **(A)** The top 10 significantly enriched GO BP terms with *P* < 0.1 of candidate DmRNAs. Significant enrichment results for **(B)** upregulated and **(C)** downregulated mRNAs are shown, respectively. Gene count: the number of DmRNAs enriched in the GO term. Gene ratio: the proportion of DmRNAs enriched in the GO term to the total DmRNAs. P.Value: the enrichment significance p-value. BP: biological process.

Non-coding RNAs often function by regulating the expression of protein-coding mRNAs, involved in various biological processes. We performed functional enrichment analysis on candidate DmRNAs using DAVID software, separately for upregulated and downregulated mRNAs in CDDP-resistant LUAD cell lines. The analysis results showed that these DmRNAs were significantly enriched in multiple GO biological process (BP) terms, and by reviewing reliable publications, we found that the most significantly enriched GO functions (Fig. 2A) were closely associated with the occurrence, progression, and treatment response of LUAD. For example, the significant GO functions of upregulated mRNAs included cell adhesion 32, sprouting angiogenesis 33, response to hypoxia 34, 35, positive regulation of protein phosphorylation 36 (Fig. 2B), and the significant GO functions of downregulated mRNAs included positive regulation of apoptotic process 37, 38, activation of cysteine-type endopeptidase activity involved in apoptotic process 37 (Fig. 2C).

### Identification of ceRNA interactions associated with CDDP resistance in LUAD

CDDP resistance is a complex process, so we cannot attribute it to a single factor. Dysregulation of ceRNA interactions may be one of the mechanisms leading to CDDP resistance. To further understand the roles of DlncRNAs, DmiRNAs and DmRNAs in CDDP resistance in LUAD and to clarify the relationships among them, we systematically identified functional DlncRNA-DmiRNA-DmRNA competing triplets by incorporating miRNA-binding prediction algorithm, experimental support, and expression correlation model.

**Figure 3:**
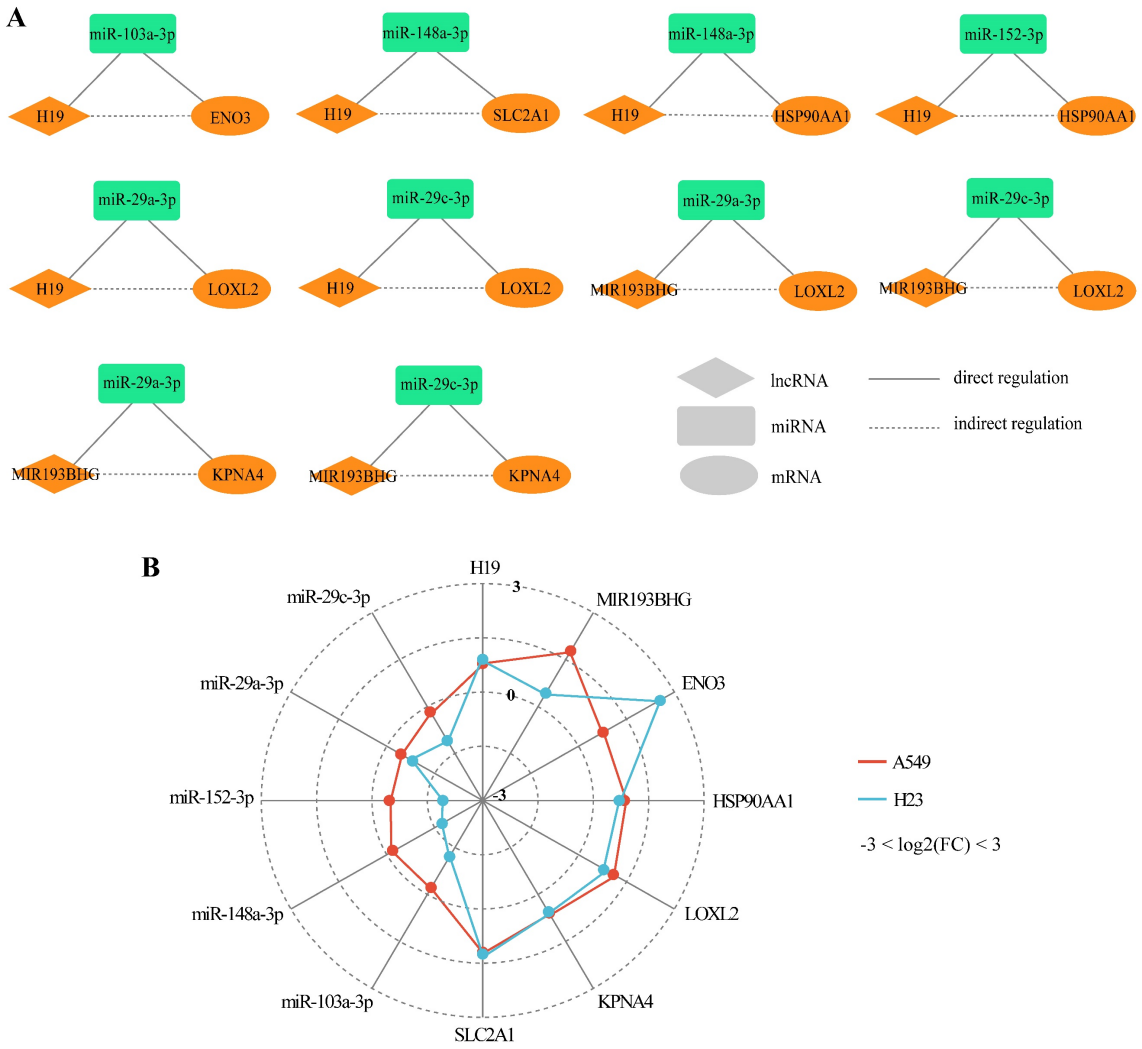
LncRNA-mediated competing triplets associated with CDDP resistance in LUAD exhibit variable competing patterns in resistant cells compared to matched sensitive cells. **(A)** 10 identified DlncRNA-DmiRNA-DmRNA competing triplets. Diamonds, squares and ovals represent lncRNAs, miRNAs and mRNAs, respectively. Orange and green shapes represent upregulated and downregulated molecules in CDDP-resistant LUAD cells, respectively. Solid lines represent direct interaction relationships between miRNAs and lncRNAs/mRNAs. Dashed lines connect co-expressed lncRNAs and mRNAs in indirect interaction relationships.** (B)** Differences in the expression (log2(FC) value) of each molecule in the identified competing triplets between CDDP-resistant and CDDP-sensitive LUAD cell lines, separately for A549R/S and H23R/S. Limma analysis, *P* < 0.05.

Based on the common DmiRNA interactions and co-expression correlations between DlncRNAs and DmRNAs, we identified 10 DlncRNA-DmiRNA-DmRNA competing triplets associated with CDDP resistance in LUAD (Table 1). In these lncRNA-mediated competing triplets, lncRNAs played key regulatory roles, resulting in corresponding changes in miRNA and mRNA expression. We found that their competing patterns were lncRNA-up/miRNA-down/mRNA-up in CDDP-resistant LUAD cells compared to sensitive cells (Fig. 3A), involving 2 upregulated lncRNAs (*H19*, *MIR193BHG*), 5 downregulated miRNAs (*miR-103a-3p*, *miR-148a-3p*, *miR-152-3p*, *miR-29a-3p*, *miR-29c-3p*) and 5 upregulated mRNAs (*ENO3*, *SLC2A1*, *HSP90AA1*, *LOXL2*, *KPNA4*) (Fig. 3B).

### Construction and functional annotation of the lncRNA-mediated LUAD CDDP resistance-related ceRNA network (LLCRCN)

**Figure 4:**
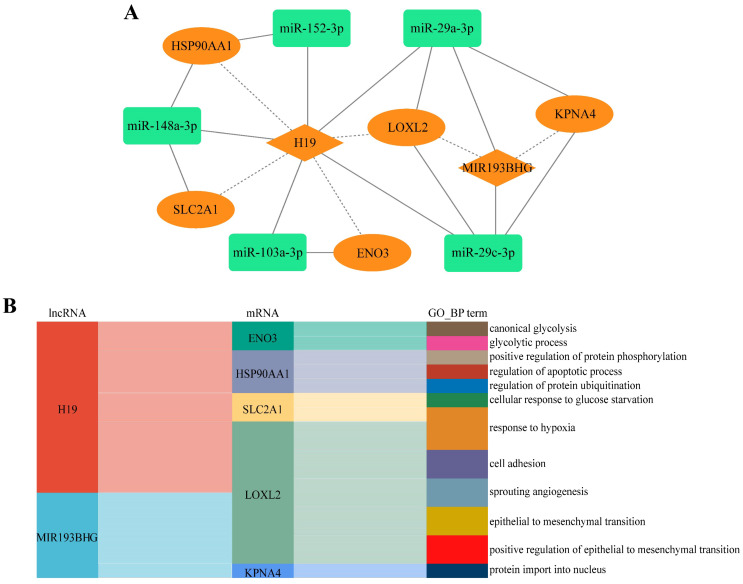
The LLCRCN is involved in biological processes related to drug response. **(A)** LLCRCN diagram. Orange and green nodes represent upregulated and downregulated lncRNAs, miRNAs or mRNAs in CDDP-resistant LUAD cells, respectively. Solid and dashed lines represent direct and indirect interactions between molecules, respectively. **(B)** The GO functional annotation of the target mRNAs indirectly regulated by the upstream lncRNAs in the LLCRCN. The colors in the Sankey diagram are used to distinguish different nodes or connection paths. The links between nodes show the associative flow relationships, which are left-to-right directions, flowing from the source nodes to the target nodes.

It was assumed that lncRNAs and mRNAs in one competing triplet could compete for binding to miRNAs within other competing triplets. Cross-talk between ceRNAs forms the competing regulatory network that plays a critical role in physiological and pathological processes 39. To further explore the ceRNA mechanism underlying CDDP resistance in LUAD, we constructed a lncRNA-mediated LUAD CDDP resistance-related ceRNA network (LLCRCN) by assembling the above identified DlncRNA-DmiRNA-DmRNA competing triplets associated with resistance, which contained 21 edges among 12 molecules (Fig. 4A).

In the LLCRCN, the upstream lncRNAs *H19* and *MIR193BHG*, acting as ceRNAs, regulated multiple miRNA-mRNA axes. The imprinted oncofetal lncRNA *H19*, which normally controls mRNA expression by acting as a miRNA sponge, has been reported to be an oncogene in a variety of cancers, including LUAD, and a powerful therapeutic target for counteracting resistance to chemotherapy and radiotherapy 40, 41. The hypoxia-inducible lncRNA *MIR193BHG*-mediated ceRNA network was reported to be one of the biological regulatory pathways in LUAD, *MIR193BHG* upregulation affected the therapeutic efficacy and was associated with poor prognosis of LUAD 42. These previous findings highlighted that the two key lncRNAs play crucial roles in LUAD through the ceRNA mechanism, which further supports our results.

Through GO functional analysis of downstream target mRNAs, the LLCRCN was identified to be involved in biological processes related to drug response, such as response to hypoxia, cell adhesion, sprouting angiogenesis, epithelial to mesenchymal transition (EMT), regulation of apoptotic process, glycolytic process (Fig. 4B). Notably, accumulating evidence has demonstrated that dysregulation of these biological processes is a major contributor to CDDP resistance 35, 43, 44.

### The LLCRCN regulates the functional states of LUAD cells

**Figure 5:**
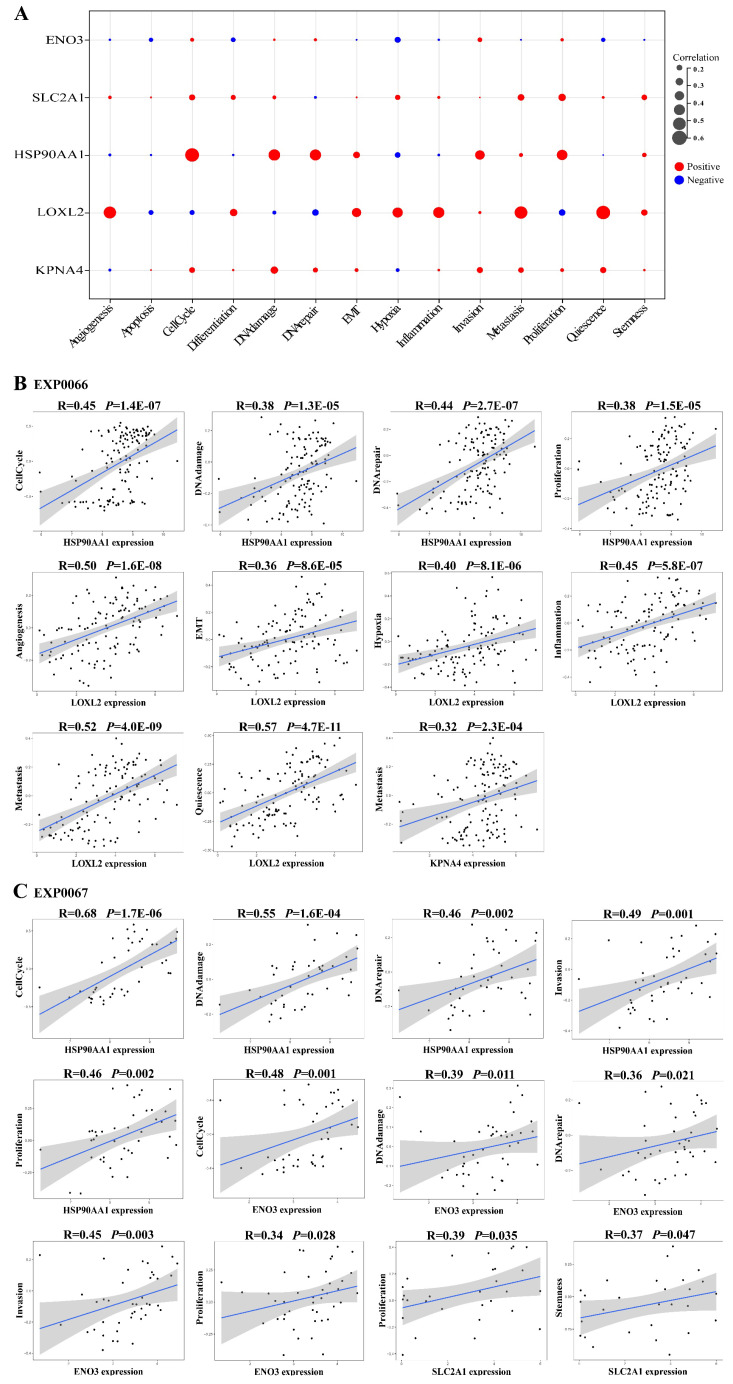
The LLCRCN regulated the functional states of LUAD cells. **(A)** Average correlation between target mRNAs in the LLCRCN and the functional states of LUAD cells in two LUAD scRNA-seq datasets. **(B)** Significant correlation results based on EXP0066 dataset. **(C)** Significant correlation results based on EXP0067 dataset. Correlation R > 0.3 and *P* < 0.05.

Cross-talk between ceRNAs represents an intricate transcriptional regulatory network that provides insight into how intermolecular relationships dictate cellular behavior. Previous studies have shown that differences in the activities of cancer cell functional states can lead to different sensitivities of cancer cells to drug treatment 45, 46. To further validate the role of the LLCRCN, we next investigated whether the LLCRCN regulates LUAD-related cellular processes. We analyzed the correlation between the expression of downstream target mRNAs in the LLCRCN and the functional state activities of LUAD cells at single-cell level based on CancerSEA data. The analysis results showed that all target mRNAs in the LLCRCN were significantly correlated with the functional states of LUAD cancer cells (Fig. 5A, average correlation in two LUAD scRNA-seq datasets). Specifically, *HSP90AA1* and *ENO3* were significantly positively correlated with cell cycle, DNA damage, DNA repair, invasion and proliferation of LUAD cancer cells; *LOXL2* was significantly positively correlated with angiogenesis, EMT, hypoxia, inflammation, metastasis and quiescence of LUAD cancer cells; *KPNA4* was significantly positively correlated with metastasis of LUAD cancer cells; *SLC2A1* was significantly positively correlated with proliferation and stemness of LUAD cancer cells (Fig. 5B-C). Collectively, these results indicated that the LLCRCN regulated the functional states of LUAD cells, further suggesting that these identified resistance-related ceRNAs may play important roles in the regulation of drug response in LUAD.

### The LLCRCN contributes to the prognosis of LUAD patients

**Figure 6:**
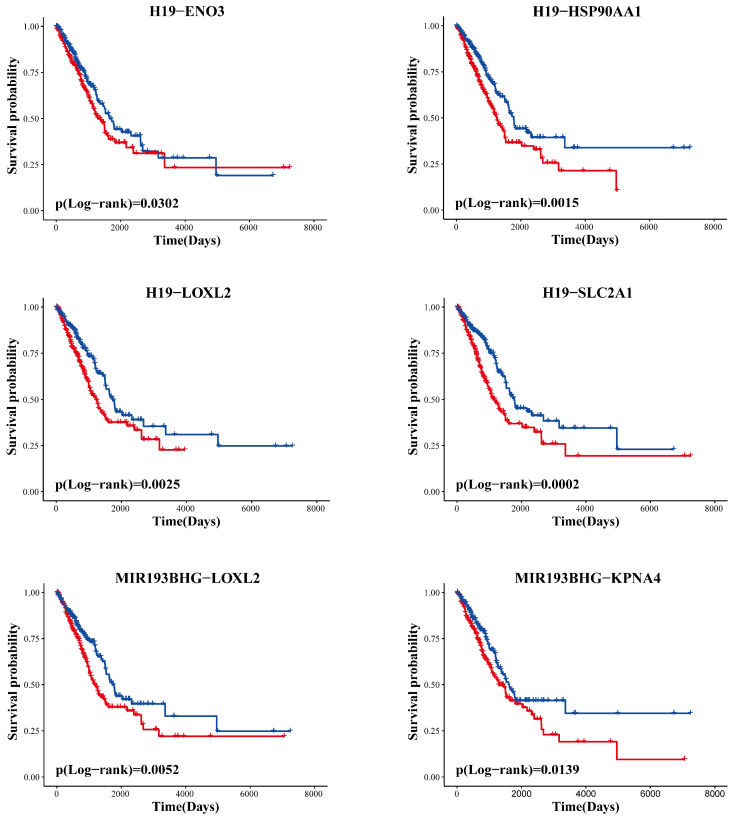
The LLCRCN is significantly associated with overall survival in LUAD patients. Clinical outcome difference between two groups classified by each ceRNA interaction pair in the LLCRCN was estimated by the Kaplan-Meier method, and the significance p-value was calculated by the log-rank test.

Drug response is generally associated with the prognosis of cancer patients. Therefore, we reasoned that if the identified ceRNAs are involved in the regulation of drug response, then they would likely be able to stratify prognosis. We performed Kaplan-Meier survival analysis to evaluate the prognostic performance of ceRNAs based on TCGA data. The analysis results showed that all ceRNA interaction pairs in the LLCRCN could successfully distinguish between two groups of LUAD patients with different clinical outcomes (Fig. 6, *P* < 0.05). These results indicated that the LLCRCN was closely associated with the prognosis of LUAD patients, further reflecting these identified resistance-related ceRNAs might act as potential signatures of drug response in LUAD.

### LncRNA regulators in the LLCRCN are correlated with CDDP activity in LUAD cell lines

**Figure 7:**
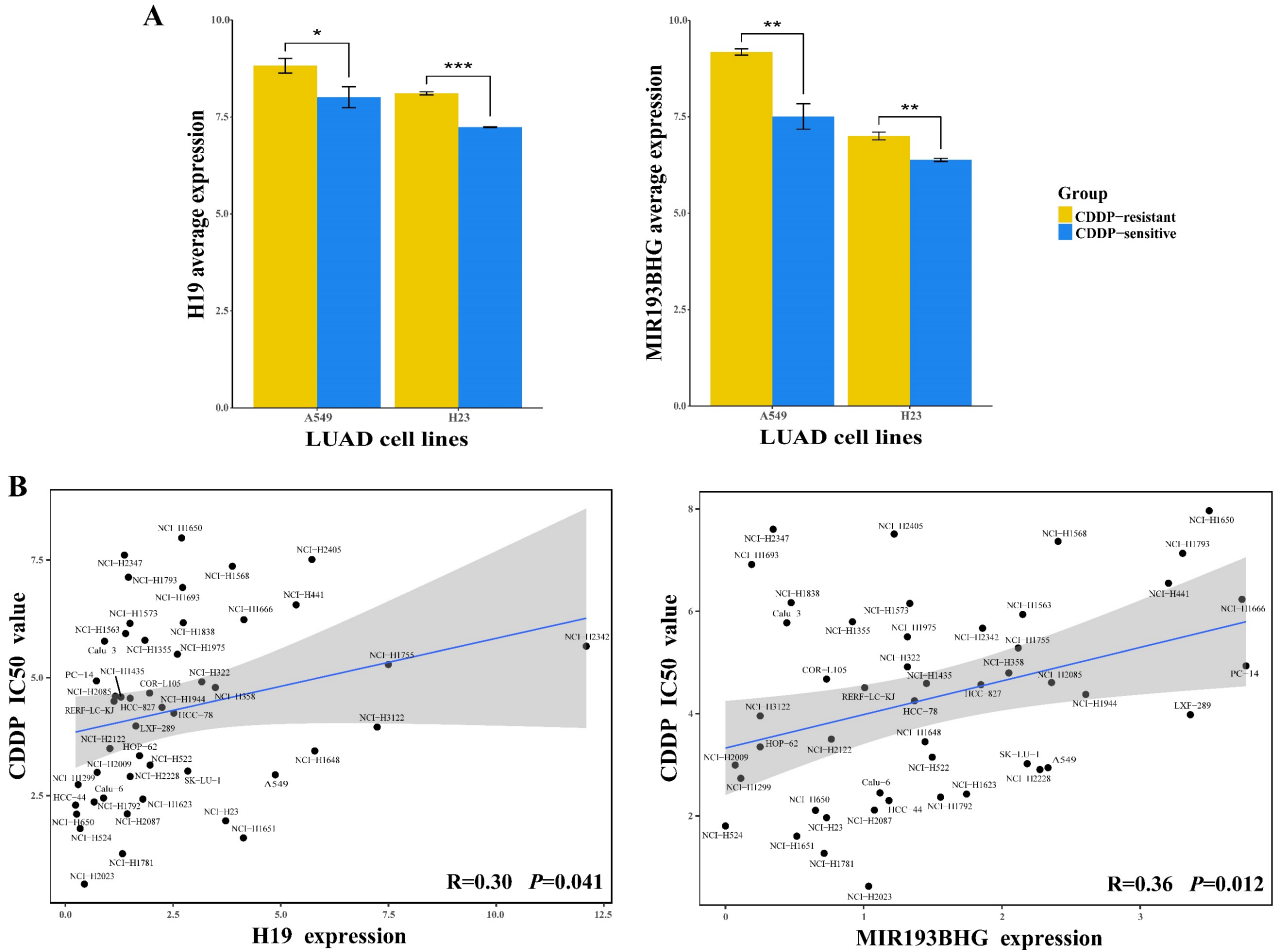
Upregulation of lncRNA regulators in the LLCRCN is significantly associated with CDDP resistance in LUAD.** (A)** Relative expression level of lncRNAs in CDDP-resistant LUAD cells and CDDP-sensitive LUAD cells, separately for A549R/S and H23R/S. Limma analysis, * *P* < 0.05, ** *P* < 0.01, *** *P* < 0.001. **(B)** Correlation between lncRNA expression and CDDP IC50 values in LUAD cell lines.

In the competing regulation, lncRNAs play prominent roles, leading to corresponding changes in miRNA and mRNA expression. Here, *H19* and *MIR193BHG* were identified as upstream lncRNA regulators in the LLCRCN, and they were found to be upregulated in CDDP-resistant LUAD cells compared to sensitive controls (Fig. 7A, FC > 1.5 and *P* < 0.05) and affected CDDP response-related functions through multiple lncRNA-miRNA-mRNA regulatory axes. To further validate the functional relevance of these key lncRNAs in drug response, we analyzed the correlation between lncRNA expression and drug activity. The expression of each lncRNA in the LLCRCN was performed by Pearson correlation analysis with CDDP IC50 values. The analysis result showed that *H19* and *MIR193BHG* were both positively correlated with CDDP IC50 values in LUAD cell lines (Fig. 7B, R > 0.3 and *P* < 0.05), indicating that high expression of these key lncRNAs may contribute to CDDP resistance in LUAD, and also further suggesting that their potential as resistance biomarkers and therapeutic targets for avoiding and reversing resistance.

### Dissection of potential ceRNA mechanisms in drug resistance

Through manual literature mining, we found that approximately 83% of the molecules in the LLCRCN have been verified to be associated with CDDP resistance in previous studies (see Supplementary Table S1), which further supports our identified results.

**Figure 8:**
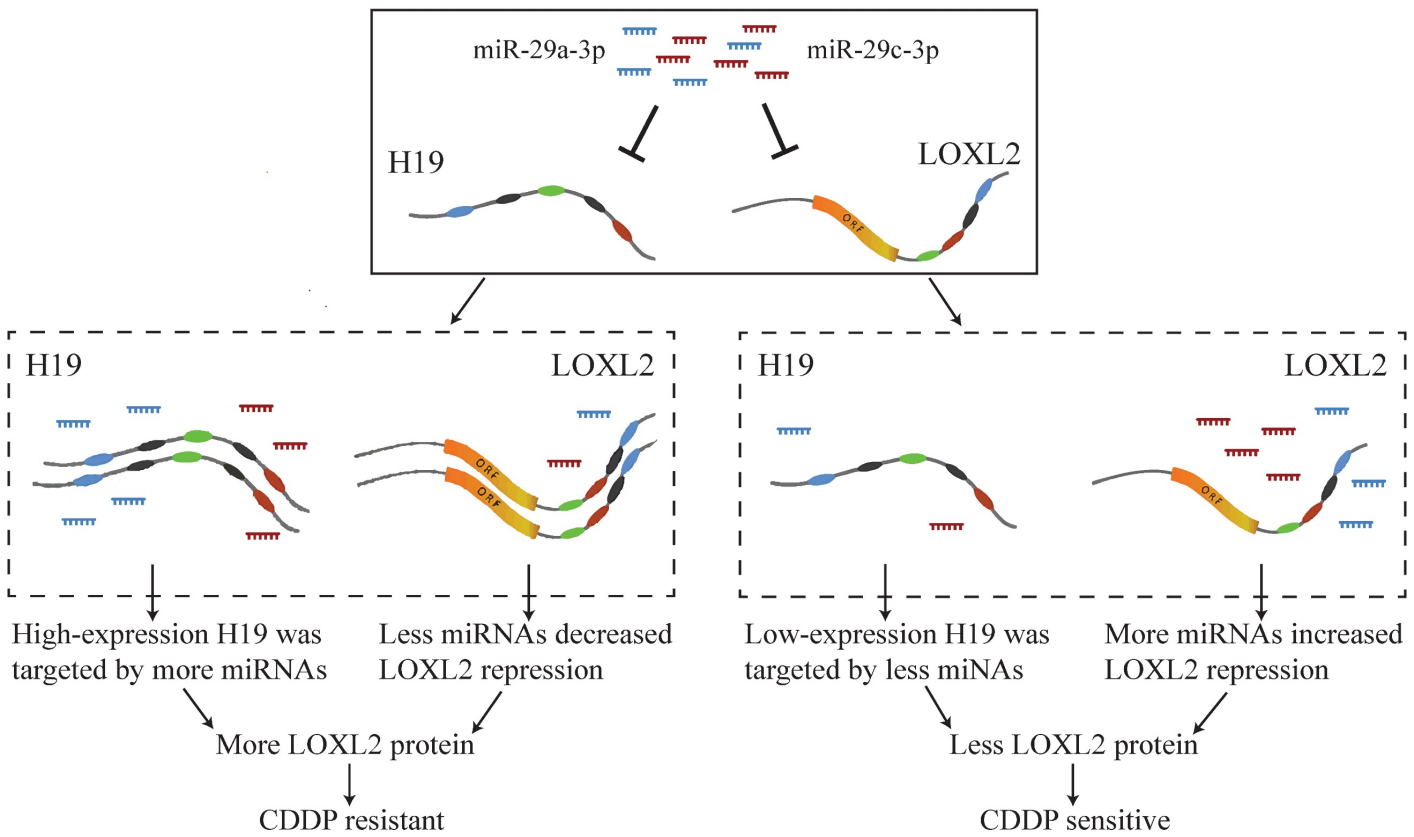
Case study of potential ceRNA mechanism of CDDP resistance in LUAD.

A previous study showed that hypoxia-induced upregulation of *H19* led to CDDP resistance in NSCLC 35. It has been reported that *miR-29a-3p* and *miR-29c-3p* act as tumor suppressors, and their downregulation can decrease the sensitivity of NSCLC cells to CDDP 47, 48. Upregulated* LOXL2* has been reported to enhance cell adhesion-dependent drug resistance 49 and to be associated with metastasis and CDDP chemoresistance of hepatocellular carcinoma 50. In this study, we identified that *H19*, acting as a ceRNA, regulated *miR-29a-3p*-*LOXL2* axis and *miR-29c-3p*-*LOXL2* axis, and the ceRNA mechanism of CDDP resistance in LUAD might be that highly expressed *H19* was targeted by more miRNAs and less free miRNAs repressed *LOXL2* expression to develop resistance (Fig. 8).

In addition, some other molecules in the LLCRCN have also been reported to be associated with CDDP resistance in lung cancer and other cancers. For example, downregulation of *miR-152-3p* and *miR-148a-3p* was found to be associated with CDDP resistance in lung cancer 20, 51 and ovarian cancer 20, 52. Upregulation of *HSP90AA1* has been shown to increase CDDP resistance in head and neck cancer 53 and osteosarcoma 54. Glycolysis-related gene *SLC2A1* was reported to be a risk factor for CDDP response in patients with LUAD and be associated with CDDP resistance 55. *KPNA4* was identified as a promoter of CDDP resistance in gastric cancer 56 and cutaneous squamous cell carcinoma 57. Our results are consistent with these previous research reports.

Therefore, based on the above results, we suggest that the LLCRCN should be involved in the regulation of CDDP resistance in LUAD. In the LLCRCN, the aberrant expression of upstream lncRNA regulators *H19* and *MIR193BHG* may result in the dysregulation of downstream target mRNA expression by competing for miRNA binding, and ultimately lead to disorders of drug response-related functions. Understanding the novel mechanisms of drug resistance allows the development of novel therapies that improve the effectiveness of chemotherapy and clinical outcomes of cancer treatment.

## Discussion

As one of the most prevalent malignancies worldwide, LUAD has already become a serious public health concern due to its poor prognosis. Although CDDP-based chemotherapy has a significant therapeutic effect on LUAD, the emergence of CDDP resistance is a major obstacle to successful treatment, and the molecular mechanisms need further investigation.

Recently, the ceRNA theory has been proposed, providing an opportunity to understand the relationships between different molecules and the regulatory mechanisms among them. Numerous studies have shown that ceRNAs take part in the regulation of cancer chemoresistance. We proposed an integrated approach to identify the regulatory roles of ceRNAs in drug resistance. We constructed a significant lncRNA-mediated ceRNA network, LLCRCN, potentially contributing to CDDP resistance in LUAD, which indicates a possible novel mechanism. The ceRNAs in the LLCRCN showed aberrant expression in multiple types of CDDP-resistant LUAD cell lines and were found to be involved in biological processes known to mediate drug response, such as cell adhesion, sprouting angiogenesis, regulation of apoptotic process, response to hypoxia. Single-cell analysis showed that these resistance-related ceRNAs were positively correlated with the functional state activities of LUAD cancer cells, such as angiogenesis, cell cycle, DNA repair, proliferation, metastasis, stemness, EMT, hypoxia. Differences in the activities of these cancer cell functional states may lead to different sensitivities of LUAD cancer cells to CDDP-based chemotherapy. These resistance-related ceRNAs were shown to be closely associated with the prognosis of LUAD patients, suggesting that the LLCRCN plays an important role in LUAD. Moreover, by reviewing reliable publications, we found that most of the molecules in the LLCRCN have been reported to be associated with CDDP resistance, and these previous findings further support our conclusions.

In this study, we found that two key lncRNAs, *H19* and *MIR193BHG*, regulate multiple miRNA-mRNA axes in the LLCRCN and correlate with CDDP activity in LUAD cell lines. *H19*, which is encoded by a gene located on human chromosome 11, is normally expressed in the embryo, but is greatly downregulated after birth and then reappears in tumors 58. Overexpression of *H19* has been shown to be associated with carcinogenesis, progression, drug resistance and poor prognosis in various types of cancer, including LUAD 40. *MIR193BHG* is a hypoxia-inducible lncRNA encoded by the *miR193b*-host gene locus on chromosome 16, and upregulation of *MIR193BHG* might affect the therapeutic efficacy and prognosis of LUAD 42. Here, we revealed the potential *H19*/*MIR193BHG*-mediated ceRNA mechanisms underlying CDDP resistance in LUAD. Our results showed that differences in individual response to CDDP might be triggered by dysregulation of ceRNA expression. When LUAD patients were treated with CDDP, resistance usually occurred. During this process, aberrant expression of lncRNA might lead to dysregulation of mRNA expression by competing for miRNA binding. Consequently, the LLCRCN was disturbed, and further affecting some hallmark functions of cancer drug resistance. That would make these key lncRNAs as potential resistance biomarkers and therapeutic targets to improve cancer treatment outcomes.

In conclusion, not only direct regulation, but also indirect stimuli, such as ceRNA, could alter individual response to chemotherapy. CeRNAs represent an additional layer of complexity in cancer chemoresistance. This study provides a valuable approach to identify functional ceRNAs associated with resistance for further experimental validation and clinical trials. Moreover, the idea of this study is not limited to dissecting the regulatory roles of ceRNAs in CDDP resistance in LUAD, but can also be applied to investigate other cancer drug resistance. To strengthen our approach, we will extend it to multidrug resistance and combine other related multi-omics data in our future study.

## Supplementary Material

Supplementary figures and tables.

## Figures and Tables

**Table 1:** The identified DlncRNA-DmRNA competing interaction pairs with shared DmiRNAs and co-expression correlations of PCC > 0.2 and *P* < 0.05.

lncRNA-mRNA	Shared miRNA	Co-expression correlation
*H19*-*ENO3*	*miR-103a-3p*	PCC=0.23, *P*=2.9E-08
*H19*-*SLC2A1*	*miR-148a-3p*	PCC=0.21, *P*=4.8E-07
*H19*-*HSP90AA1*	*miR-148a-3p*, *miR-152-3p*	PCC=0.21, *P*=7.3E-07
*H19*-*LOXL2*	*miR-29a-3p*, *miR-29c-3p*	PCC=0.31, *P*=3.2E-14
*MIR193BHG*-*LOXL2*	*miR-29a-3p*, *miR-29c-3p*	PCC=0.30, *P*=2.1E-12
*MIR193BHG*-*KPNA4*	*miR-29a-3p*, *miR-29c-3p*	PCC=0.23, *P*=8.6E-08

## Abbreviations

NSCLC: non-small cell lung cancer
LUAD: lung adenocarcinoma
CDDP: cisplatin
ceRNA: competing endogenous RNA
lncRNA: long non-coding RNA
miRNA: microRNA
mRNA: messenger RNA
MRE: miRNA response element
GEO: Gene Expression Omnibus
TCGA: The Cancer Genome Atlas
TPM: Transcripts Per Kilobase of exon model per Million mapped reads
AGO: Argonaute
CLIP-seq: crosslinking immunoprecipitation sequencing
CancerSEA: cancer single-cell state atlas
IC50: half-maximal inhibitory concentration
GDSC: Genomics of Drug Sensitivity in Cancer
CCLE: Cancer Cell Line Encyclopedia
FC: fold change
DlncRNA/DmiRNA/DmRNA: dysregulated lncRNA/miRNA/mRNA
PCC: partial correlation coefficient
GO: Gene Ontology
DAVID: Database for Annotation, Visualization and Integrated Discovery
GSVA: Gene Set Variation Analysis
BP: biological process
LLCRCN: lncRNA-mediated LUAD CDDP resistance-related ceRNA network
EMT: epithelial to mesenchymal transition

## References

[1] Siegel RL, Giaquinto AN, Jemal A (2024). Cancer statistics, 2024. CA Cancer J Clin.

[2] Kim N, Kim HK, Lee K, Hong Y, Cho JH, Choi JW (2020). Single-cell RNA sequencing demonstrates the molecular and cellular reprogramming of metastatic lung adenocarcinoma. Nat Commun.

[3] Heigener DF, Kerr KM, Laing GM, Mok TSK, Moiseyenko FV, Reck M (2019). Redefining Treatment Paradigms in First-line Advanced Non-Small-Cell Lung Cancer. Clin Cancer Res.

[4] Galluzzi L, Vitale I, Michels J, Brenner C, Szabadkai G, Harel-Bellan A (2014). Systems biology of cisplatin resistance: past, present and future. Cell Death Dis.

[5] Makovec T (2019). Cisplatin and beyond: molecular mechanisms of action and drug resistance development in cancer chemotherapy. Radiol Oncol.

[6] Wei X, Jiang Y, Yang G, Chang T, Sun G, Chen S (2023). MicroRNA-367-3p directly targets RAB23 and inhibits proliferation, migration and invasion of bladder cancer cells and increases cisplatin sensitivity. J Cancer Res Clin Oncol.

[7] Fan CC, Tsai ST, Lin CY, Chang LC, Yang JC, Chen GY (2020). EFHD2 contributes to non-small cell lung cancer cisplatin resistance by the activation of NOX4-ROS-ABCC1 axis. Redox Biol.

[8] Wang L, Ma L, Xu F, Zhai W, Dong S, Yin L (2018). Role of long non-coding RNA in drug resistance in non-small cell lung cancer. Thorac Cancer.

[9] Braga EA, Fridman MV, Burdennyy AM, Loginov VI, Dmitriev AA, Pronina IV (2023). Various LncRNA Mechanisms in Gene Regulation Involving miRNAs or RNA-Binding Proteins in Non-Small-Cell Lung Cancer: Main Signaling Pathways and Networks. Int J Mol Sci.

[10] Herman AB, Tsitsipatis D, Gorospe M (2022). Integrated lncRNA function upon genomic and epigenomic regulation. Mol Cell.

[11] Salmena L, Poliseno L, Tay Y, Kats L, Pandolfi PP (2011). A ceRNA hypothesis: the Rosetta Stone of a hidden RNA language?. Cell.

[12] Wang P, Zhi H, Zhang Y, Liu Y, Zhang J, Gao Y (2015). miRSponge: a manually curated database for experimentally supported miRNA sponges and ceRNAs. Database (Oxford).

[13] Li W, Liu Y, Li ZJ, Shi Y, Deng J, Bai J (2021). Unravelling the Role of LncRNA WT1-AS/miR-206/NAMPT Axis as Prognostic Biomarkers in Lung Adenocarcinoma. Biomolecules.

[14] Wang P, Li X, Gao Y, Guo Q, Wang Y, Fang Y (2019). LncACTdb 2.0: an updated database of experimentally supported ceRNA interactions curated from low- and high-throughput experiments. Nucleic Acids Res.

[15] Qu L, Ding J, Chen C, Wu ZJ, Liu B, Gao Y (2016). Exosome-Transmitted lncARSR Promotes Sunitinib Resistance in Renal Cancer by Acting as a Competing Endogenous RNA. Cancer Cell.

[16] Liu H, Wang S, Zhou S, Meng Q, Ma X, Song X (2019). Drug Resistance-Related Competing Interactions of lncRNA and mRNA across 19 Cancer Types. Mol Ther Nucleic Acids.

[17] Wu A, Liu J, Zhang X, Niu C, Shu G, Yin G (2022). Comprehensive network analysis of dysregulated genes revealed MNX1-AS1/hsa-miR-4697-3p/HOXB13 axis in ovarian cancer chemotherapy response. Cancer Sci.

[18] Yang Y, Li H, Hou S, Hu B, Liu J, Wang J (2013). The noncoding RNA expression profile and the effect of lncRNA AK126698 on cisplatin resistance in non-small-cell lung cancer cell. Plos One.

[19] Vera O, Rodriguez-Antolin C, de Castro J, Karreth FA, Sellers TA, Ibanez de Caceres I (2018). An epigenomic approach to identifying differential overlapping and cis-acting lncRNAs in cisplatin-resistant cancer cells. Epigenetics-Us.

[20] Vera O, Jimenez J, Pernia O, Rodriguez-Antolin C, Rodriguez C, Sanchez Cabo F (2017). DNA Methylation of miR-7 is a Mechanism Involved in Platinum Response through MAFG Overexpression in Cancer Cells. Theranostics.

[21] Derrien T, Johnson R, Bussotti G, Tanzer A, Djebali S, Tilgner H (2012). The GENCODE v7 catalog of human long noncoding RNAs: analysis of their gene structure, evolution, and expression. Genome Res.

[22] Kozomara A, Griffiths-Jones S (2014). miRBase: annotating high confidence microRNAs using deep sequencing data. Nucleic Acids Res.

[23] Li JH, Liu S, Zhou H, Qu LH, Yang JH (2014). starBase v2.0: decoding miRNA-ceRNA, miRNA-ncRNA and protein-RNA interaction networks from large-scale CLIP-Seq data. Nucleic Acids Res.

[24] Karagkouni D, Paraskevopoulou MD, Chatzopoulos S, Vlachos IS, Tastsoglou S, Kanellos I (2018). DIANA-TarBase v8: a decade-long collection of experimentally supported miRNA-gene interactions. Nucleic Acids Res.

[25] Huang HY, Lin YC, Cui S, Huang Y, Tang Y, Xu J (2022). miRTarBase update 2022: an informative resource for experimentally validated miRNA-target interactions. Nucleic Acids Res.

[26] Yuan H, Yan M, Zhang G, Liu W, Deng C, Liao G (2019). CancerSEA: a cancer single-cell state atlas. Nucleic Acids Res.

[27] Ritchie ME, Phipson B, Wu D, Hu Y, Law CW, Shi W (2015). limma powers differential expression analyses for RNA-sequencing and microarray studies. Nucleic Acids Res.

[28] Wang P, Ning S, Zhang Y, Li R, Ye J, Zhao Z (2015). Identification of lncRNA-associated competing triplets reveals global patterns and prognostic markers for cancer. Nucleic Acids Res.

[29] Yoshihara K, Shahmoradgoli M, Martinez E, Vegesna R, Kim H, Torres-Garcia W (2013). Inferring tumour purity and stromal and immune cell admixture from expression data. Nat Commun.

[30] Huang da W, Sherman BT, Lempicki RA (2009). Systematic and integrative analysis of large gene lists using DAVID bioinformatics resources. Nat Protoc.

[31] Hanzelmann S, Castelo R, Guinney J (2013). GSVA: gene set variation analysis for microarray and RNA-seq data. Bmc Bioinformatics.

[32] Komori Y, Kano J, Nakano N, Sakashita S, Sakamoto N, Noguchi M (2019). Dickkopf-related protein 3 promotes cell adhesion and invasion during progression of lung adenocarcinoma. Pathol Int.

[33] Cai S, Guo X, Huang C, Deng Y, Du L, Liu W (2021). Integrative analysis and experiments to explore angiogenesis regulators correlated with poor prognosis, immune infiltration and cancer progression in lung adenocarcinoma. J Transl Med.

[34] Zhao M, Zhang Y, Zhang H, Wang S, Zhang M, Chen X (2015). Hypoxia-induced cell stemness leads to drug resistance and poor prognosis in lung adenocarcinoma. Lung Cancer.

[35] Li H, Wang J, Jin Y, Lin J, Gong L, Xu Y (2022). Hypoxia upregulates the expression of lncRNA H19 in non-small cell lung cancer cells and induces drug resistance. Transl Cancer Res.

[36] Du R, Wang C, Liu J, Wang K, Dai L, Shen W (2023). Phosphorylation of TGIF2 represents a therapeutic target that drives EMT and metastasis of lung adenocarcinoma. BMC Cancer.

[37] Johnstone RW, Ruefli AA, Lowe SW (2002). Apoptosis: a link between cancer genetics and chemotherapy. Cell.

[38] Zheng QW, Ni QZ, Zhu B, Liang X, Ma N, Wang YK (2022). PPDPF promotes lung adenocarcinoma progression via inhibiting apoptosis and NK cell-mediated cytotoxicity through STAT3. Oncogene.

[39] Sanchez-Mejias A, Tay Y (2015). Competing endogenous RNA networks: tying the essential knots for cancer biology and therapeutics. J Hematol Oncol.

[40] Yang J, Qi M, Fei X, Wang X, Wang K (2021). LncRNA H19: A novel oncogene in multiple cancers. Int J Biol Sci.

[41] Garcia-Padilla C, Lozano-Velasco E, Munoz-Gallardo MDM, Castillo-Casas JM, Cano-Carrillo S, Martinez-Amaro FJ (2022). LncRNA H19 Impairs Chemo and Radiotherapy in Tumorigenesis. Int J Mol Sci.

[42] Zhou Q, Li D, Zheng H, He Z, Qian F, Wu X (2021). A novel lncRNA-miRNA-mRNA competing endogenous RNA regulatory network in lung adenocarcinoma and kidney renal papillary cell carcinoma. Thorac Cancer.

[43] Wang MR, Chen RJ, Zhao F, Zhang HH, Bi QY, Zhang YN (2020). Effect of Wenxia Changfu Formula Combined With Cisplatin Reversing Non-Small Cell Lung Cancer Cell Adhesion-Mediated Drug Resistance. Front Pharmacol.

[44] Cheng Q, Zhou L, Zhou J, Wan H, Li Q, Feng Y (2016). ACE2 overexpression inhibits acquired platinum resistance-induced tumor angiogenesis in NSCLC. Oncol Rep.

[45] Giustacchini A, Thongjuea S, Barkas N, Woll PS, Povinelli BJ, Booth CAG (2017). Single-cell transcriptomics uncovers distinct molecular signatures of stem cells in chronic myeloid leukemia. Nat Med.

[46] Chen M, Zhang S, Wang F, He J, Jiang W, Zhang L (2024). DLGAP5 promotes lung adenocarcinoma growth via upregulating PLK1 and serves as a therapeutic target. J Transl Med.

[47] Chen X, Zhu H, Ye W, Cui Y, Chen M (2019). MicroRNA-29a enhances cisplatin sensitivity in non-small cell lung cancer through the regulation of REV3L. Mol Med Rep.

[48] Sun DM, Tang BF, Li ZX, Guo HB, Cheng JL, Song PP (2018). MiR-29c reduces the cisplatin resistance of non-small cell lung cancer cells by negatively regulating the PI3K/Akt pathway. Sci Rep.

[49] Gong L, Zhang Y, Yang Y, Yan Q, Ren J, Luo J (2022). Inhibition of lysyl oxidase-like 2 overcomes adhesion-dependent drug resistance in the collagen-enriched liver cancer microenvironment. Hepatol Commun.

[50] Leung MS, Chan KK, Dai WJ, Wong CY, Au KY, Wong PY (2020). Anti-tumour effects of PIM kinase inhibition on progression and chemoresistance of hepatocellular carcinoma. J Pathol.

[51] Zhao L, Wu X, Zhang Z, Fang L, Yang B, Li Y (2023). ELF1 suppresses autophagy to reduce cisplatin resistance via the miR-152-3p/NCAM1/ERK axis in lung cancer cells. Cancer Sci.

[52] Khajehnoori S, Zarei F, Mazaheri M, Dehghani-Firoozabadi A (2020). Epidrug Modulated Expression of MiR-152 and MiR-148a Reverse Cisplatin Resistance in Ovarian Cancer Cells: An Experimental In-vitro Study. Iran J Pharm Res.

[53] Ling J, Zhang L, Wang Y, Chang A, Huang Y, Zhao H (2023). Fisetin, a dietary flavonoid, increases the sensitivity of chemoresistant head and neck carcinoma cells to cisplatin possibly through HSP90AA1/IL-17 pathway. Phytother Res.

[54] Xiao X, Wang W, Li Y, Yang D, Li X, Shen C (2018). HSP90AA1-mediated autophagy promotes drug resistance in osteosarcoma. J Exp Clin Cancer Res.

[55] Zhao R, Ding D, Ding Y, Han R, Wang X, Zhu C (2022). Predicting Differences in Treatment Response and Survival Time of Lung Adenocarcinoma Patients Based on a Prognostic Risk Model of Glycolysis-Related Genes. Front Genet.

[56] Sun H, Zhou R, Zheng Y, Wen Z, Zhang D, Zeng D (2021). CRIP1 cooperates with BRCA2 to drive the nuclear enrichment of RAD51 and to facilitate homologous repair upon DNA damage induced by chemotherapy. Oncogene.

[57] Zhang M, Luo H, Hui L (2019). MiR-3619-5p hampers proliferation and cisplatin resistance in cutaneous squamous-cell carcinoma via KPNA4. Biochem Biophys Res Commun.

[58] Raveh E, Matouk IJ, Gilon M, Hochberg A (2015). The H19 Long non-coding RNA in cancer initiation, progression and metastasis - a proposed unifying theory. Mol Cancer.

