# Supplementary material for: A Novel Competing Endogenous RNA Network Reveals Potential Mechanisms and Biomarkers of Chemoresistance in Lung Adenocarcinoma
Source: J Cancer. 2025 Jan 1;16(3):720–34. doi: 10.7150/jca.102148 (PMC11705065; doi:10.7150/jca.102148)
Supplement: Supplementary file 1 — Supplementary figures and tables. [file jcav16p0720s1.pdf]

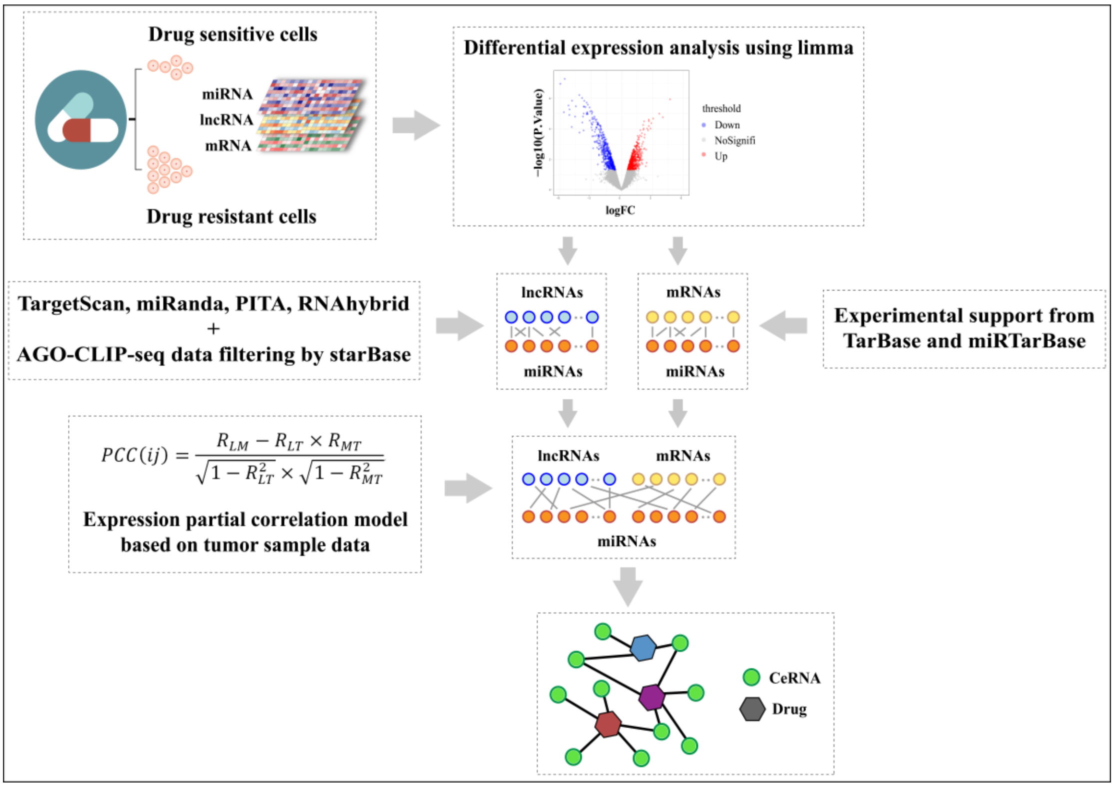

Supplementary Figure S1. Schematic workflow of the multi-step pipeline for the identification of functional ceRNAs associated with drug resistance.

Table S1. Summary of molecules in the LLCRCN that have been reported to be associated with CDDP resistance in lung cancer and other cancers.

Molecules in the LLCRCN	Associations with CDDP resistance in cancer reported in previous studies
H19	non-small cell lung cancer [1], ovarian cancer [2], laryngeal squamous cell carcinoma [3]
miR-103a-3p	osteosarcoma [4]
miR-148a-3p	lung cancer [5], ovarian cancer [5, 6], cervical cancer [7], gastric cancer [8]
miR-152-3p	lung cancer [9], ovarian cancer [6]
miR-29a-3p	non-small cell lung cancer [10, 11], ovarian cancer [12]
miR-29c-3p	non-small cell lung cancer [13], ovarian cancer [12, 14]
SLC2A1	lung adenocarcinoma [15]
HSP90AA1	head and neck cancer [16], osteosarcoma [17]
LOXL2	hepatocellular carcinoma [18]
KPNA4	gastric cancer [19], cutaneous squamous cell carcinoma [20]

References

1. Li H, Wang J, Jin Y, Lin J, Gong L, Xu Y. Hypoxia upregulates the expression of lncRNA H19 in non-small cell lung cancer cells and induces drug resistance. Transl Cancer Res. 2022; 11: 2876-86.

2. Wu Y, Zhou Y, He J, Sun H, Jin Z. Long non-coding RNA H19 mediates ovarian cancer cell cisplatin-resistance and migration during EMT. Int J Clin Exp Pathol. 2019; 12: 2506-15.

3. Chen L, Xu Z, Zhao J, Zhai X, Li J, Zhang Y, et al. H19/miR-107/HMGB1 axis sensitizes laryngeal squamous cell carcinoma to cisplatin by suppressing autophagy in vitro and in vivo. Cell Biol Int. 2021; 45: 674-85.

4. Huang JW, Wang Y, Dhillon KK, Calses P, Villegas E, Mitchell PS, et al. Systematic screen identifies miRNAs that target RAD51 and RAD51D to enhance chemosensitivity. Mol Cancer Res. 2013; 11: 1564-73.

5. Vera O, Jimenez J, Pernia O, Rodriguez-Antolin C, Rodriguez C, Sanchez Cabo F, et al. DNA Methylation of miR-7 is a Mechanism Involved in Platinum Response through MAFG Overexpression in Cancer Cells. Theranostics. 2017; 7: 4118-34.

6. Khajehnoori S, Zarei F, Mazaheri M, Dehghani-Firoozabadi A. Epidrug Modulated Expression of MiR--152 and MiR-148a Reverse Cisplatin Resistance in Ovarian Cancer Cells: An Experimental In-vitro Study. Iran J Pharm Res. 2020; 19: 509-19.

7. Yang H, Hou H, Zhao H, Yu T, Hu Y, Hu Y, et al. HK2 Is a Crucial Downstream Regulator of miR-148a for the Maintenance of Sphere-Forming Property and Cisplatin Resistance in Cervical Cancer Cells. Front Oncol. 2021; 11: 794015.

8. Li B, Wang W, Li Z, Chen Z, Zhi X, Xu J, et al. MicroRNA-148a-3p enhances cisplatin cytotoxicity in gastric cancer through mitochondrial fission induction and cyto-protective autophagy suppression. Cancer Lett. 2017; 410: 212-27.

9. Zhao L, Wu X, Zhang Z, Fang L, Yang B, Li Y. ELF1 suppresses autophagy to reduce cisplatin resistance via the miR-152-3p/NCAM1/ERK axis in lung cancer cells. Cancer Sci. 2023; 114: 2650-63.
10. Chen X, Zhu H, Ye W, Cui Y, Chen M. MicroRNA-29a enhances cisplatin sensitivity in non-small cell lung cancer through the regulation of REV3L. Mol Med Rep. 2019; 19: 831-40.
11. Liu X, Lv X, Yang Q, Jin H, Zhou W, Fan Q. MicroRNA-29a Functions as a Tumor Suppressor and Increases Cisplatin Sensitivity by Targeting NRAS in Lung Cancer. Technol Cancer Res Treat. 2018; 17: 1533033818758905.
12. Yu PN, Yan MD, Lai HC, Huang RL, Chou YC, Lin WC, et al. Downregulation of miR-29 contributes to cisplatin resistance of ovarian cancer cells. Int J Cancer. 2014; 134: 542-51.
13. Sun DM, Tang BF, Li ZX, Guo HB, Cheng JL, Song PP, et al. MiR-29c reduces the cisplatin resistance of non-small cell lung cancer cells by negatively regulating the PI3K/Akt pathway. Sci Rep. 2018; 8: 8007.
14. Hu Z, Cai M, Zhang Y, Tao L, Guo R. miR-29c-3p inhibits autophagy and cisplatin resistance in ovarian cancer by regulating FOXP1/ATG14 pathway. Cell Cycle. 2020; 19: 193-206.
15. Zhao R, Ding D, Ding Y, Han R, Wang X, Zhu C. Predicting Differences in Treatment Response and Survival Time of Lung Adenocarcinoma Patients Based on a Prognostic Risk Model of Glycolysis-Related Genes. Front Genet. 2022; 13: 828543.
16. Ling J, Zhang L, Wang Y, Chang A, Huang Y, Zhao H, et al. Fisetin, a dietary flavonoid, increases the sensitivity of chemoresistant head and neck carcinoma cells to cisplatin possibly through HSP90AA1/IL-17 pathway. Phytother Res. 2023; 37: 1997-2011.
17. Xiao X, Wang W, Li Y, Yang D, Li X, Shen C, et al. HSP90AA1-mediated autophagy promotes drug resistance in osteosarcoma. J Exp Clin Cancer Res. 2018; 37: 201.
18. Leung MS, Chan KK, Dai WJ, Wong CY, Au KY, Wong PY, et al. Anti-tumour effects of PIM kinase inhibition on progression and chemoresistance of hepatocellular carcinoma. J Pathol. 2020; 252: 65-76.
19. Sun H, Zhou R, Zheng Y, Wen Z, Zhang D, Zeng D, et al. CRIP1 cooperates with BRCA2 to drive the nuclear enrichment of RAD51 and to facilitate homologous repair upon DNA damage induced by chemotherapy. Oncogene. 2021; 40: 5342-55.
20. Zhang M, Luo H, Hui L. MiR-3619-5p hampers proliferation and cisplatin resistance in cutaneous squamous-cell carcinoma via KPNA4. Biochem Biophys Res Commun. 2019; 513: 419-25.
